# A_3 _adenosine receptors and mitogen-activated protein kinases in lung injury following *in vivo *reperfusion

**DOI:** 10.1186/cc4893

**Published:** 2006-04-19

**Authors:** Idit Matot, Carolyn F Weiniger, Evelyne Zeira, Eithan Galun, Bhalchandra V Joshi, Kenneth A Jacobson

**Affiliations:** 1 Department of Anesthesiology & Critical Care Medicine, Hadassah University Medical Center, The Hebrew University, Jerusalem, Israel; 2 Goldyne Savad Institute of Gene Therapy, Hadassah University Medical Center, The Hebrew University, Jerusalem, Israel; 3 Molecular Recognition Section, Laboratory of Bioorganic Chemistry, National Institute of Diabetes, Digestive and Kidney Diseases, National Institutes of Health, Bethesda, Maryland, USA

## Abstract

**Introduction:**

Although activation of A_3 _adenosine receptors attenuates reperfusion lung injury and associated apoptosis, the signaling pathway that mediates this protection remains unclear. Adenosine agonists activate mitogen-activated protein kinases, and these kinases have been implicated in ischemia/reperfusion injury; the purpose of this study was therefore to determine whether A_3 _adenosine receptor stimulation with reperfusion modulates expression of the different mitogen-activated protein kinases. In addition, we compared the effect of the A_3 _adenosine agonist IB-MECA with the newly synthesized, highly selective A_3 _adenosine receptor agonist MRS3558 on injury in reperfused lung.

**Method:**

Studies were performed in an *in vivo *spontaneously breathing cat model, in which the left lower lobe of the lung was isolated and subjected to 2 hours of ischemia and 3 hours of reperfusion. The selective A_3 _adenosine receptor agonists IB-MECA (0.05 mg/kg, 0.1 mg/kg, or 0.3 mg/kg) and MRS3558 (0.05 mg/kg or 0.1 mg/kg) were administered before reperfusion.

**Results:**

Both A_3 _adenosine receptor agonists administered before reperfusion markedly (*P *< 0.01) attenuated indices of injury and apoptosis, including the percentage of injured alveoli, wet/dry weight ratio, myeloperoxidase activity, TUNEL (*in situ *TdT-mediated dUTP nick end labeling)-positive cells, and caspase 3 activity and expression. The more pronounced effects at low doses were observed with MRS3558. Increases in phosphorylated c-Jun amino-terminal protein kinase (JNK), p38, and extracellular signal-regulated kinase (ERK)1/2 levels were observed by the end of reperfusion compared with controls. Pretreatment with the A_3 _agonists upregulated phosphorylated ERK1/2 levels but did not modify phosphorylated JNK and p38 levels.

**Conclusion:**

The protective effects of A_3 _adenosine receptor activation are mediated in part through upregulation of phosphorylated ERK. Also, MRS3558 was found to be more potent than IB-MECA in attenuating reperfusion lung injury. The results suggest not only that enhancement of the ERK pathway may shift the balance between cell death and survival toward cell survival, but also that A_3 _agonists have potential as an effective therapy for ischemia/reperfusion-induced lung injury.

## Introduction

Despite refinements in lung preservation and improvements in surgical techniques and perioperative care, ischemia/reperfusion (IR)-induced lung injury remains a significant cause of early morbidity and mortality after lung transplantation [1]. Pulmonary dysfunction following reperfusion has also been reported after warm ischemia in situations such as pulmonary thromboembolectomy, thrombolysis, and cardiopulmonary bypass [2]. Novel therapeutic interventions for attenuation of IR lung injury remain a focus of intense research.

Mitogen-activated protein kinases (MAPKs) are serine-threonine protein kinases that participate in anti-inflammatory/inflammatory cell signaling and the trauma and disorders associated with these processes, such as hemorrhagic shock and IR injury [3,4]. Three major MAPK families have been identified: the extracellular signal-regulated kinases (ERKs), the c-Jun amino-terminal protein kinases (JNKs) and the p38 kinases. MAPKs have been shown to be activated in animal models of reperfusion injury [3-7], suggesting that these kinases are potential targets for attenuation of IR injury [8-11].

Four different adenosine receptors (ARs) have been identified and pharmacologically characterized, namely A_1_, A_2A_, A_2B_, and A_3 _[12]. In addition to coupling to classical second messenger pathways such as modulation of cAMP production, the ARs couple to MAPKs [13]. Despite numerous reports evaluating the signaling from ARs to MAPKs [13], only a few studies have investigated this relationship during reperfusion injury, all of which were conducted in heart [14,15]. The association between A_3_AR, MAPKs, and IR lung injury has not previously been evaluated and is the focus of the present study.

The A_3_AR subtype is the most recently characterized member of the AR family [16,17] and has been a subject scrutiny in relation to potential therapeutic approaches for treating inflammatory and neurodegenerative diseases and cancer [18-21]. We previously demonstrated that selective activation of the A_3_AR subtype with reperfusion attenuated IR lung injury and associated apoptosis [22,23]. The effect of A_3_AR on MAPK during *in vivo *IR injury of the lung has not yet been reported.

Recently, a new class of A_3_AR agonists with high affinity and selectivity was designed. Specifically, enhancement of affinity at the A_3_AR relative to the other AR subtypes was achieved with the (*N*)-methanocarba substitution of the ribose ring. However, the (*N*)-methanocarba substitution alone appeared to reduce A_3_AR efficacy; the addition of 5'-N-methyluronamido moiety preserved full agonism without reducing the associated A_3_AR selectivity [24,25]. MRS3558 [(1'R,2'R,3'S,4'R,5'S)-4-{2-chloro-6-[(3-chlorophenylmethyl)amino]purin-9-yl}-1-(methylaminocarbonyl)bicyclo [3.1.0]hexane-2,3-diol] is a newly synthesized A_3_AR analog, which belongs to this series of (*N*)-methanocarba nucleosides. It is 1000-fold more selective for the A_3_AR than for the A_1_, A_2A_, and A_2B _subtypes, and the presence of a 5'-N-methyluronamido moiety endows it with full agonism [25].

The objectives of the present study were threefold: first, to evaluate MAPK activation in the reperfused lung; second, to determine whether the protective effects of A_3_AR activation on lung injury are mediated, in part, by modulation of the MAPK pathway; and third, to compare the effects of the highly selective A_3_AR agonist MRS3558 with those of the moderately selective A_3_AR nucleoside IB-MECA (N6-(3-iodobenzyl)-N-methyl-5'-carbamoyladenosine) on lung injury and apoptosis in an *in vivo *IR model.

## Materials and methods

### Animals

Adult cats weighing 2.5 to 3.5 kg were used in this investigation. All experiments were performed in accordance with the guidelines of the Animal Care and Use Committee of the Hebrew University-Hadassah School of Medicine, and with approval of the Institutional Animal Care and Use Committee.

### Materials

All chemicals were obtained from Sigma (Sigma-Aldrich Israel Ltd., Rehovot, Israel) unless specified otherwise. IB-MECA and MRS1191 were purchased from Sigma RBI (Natick, MA, USA). MRS3558 was prepared as described elsewhere [25].

### *In vivo *animal model

A standard reperfusion lung model of injury in intact chest, spontaneously breathing cat was employed, as described previously in detail [22,23,26,27]. Briefly, cats were anesthetized with barbital and, with the aid of fluoroscopy, a specially designed 6F triple-lumen catheter was advanced from the left external jugular vein into the lobar artery of the left lower lobe (LLL). Also with the use of fluoroscopy, a 4F bronchial blocker was inserted into the LLL bronchus. After heparinization the LLL was perfused at 35 ml/minute with blood withdrawn from the aorta through a catheter in the femoral artery using a Harvard peristaltic pump. The LLL was isolated by distending a balloon with contrast dye on the LLL arterial catheter. After a 1 hour period of stabilization, ischemia of the LLL was induced by discontinuing the Harvard peristaltic pump for 2 hours (ischemic period), and the perfusion circuit was then attached to a femoral vein catheter. The balloon on the tip of the bronchial blocker was distended with contrast dye to block ventilation to the lobe. After 2 hours of ischemia, the balloon on the bronchial blocker was deflated, the perfusion circuit was reattached to the arterial catheter in the LLL, and the LLL was reperfused (reperfusion period) for 3 hours at a rate of 35 ml/minute, using a Harvard peristaltic pump (as described above). In all groups, hemodynamic measurements and arterial blood gases were obtained before ischemia, after 1 hour and 2 hours of ischemia, and after 1 hour and 3 hours of reperfusion.

After 3 hours of reperfusion, animals received an overdose of pentobarbital sodium (30 mg/kg). Lung tissue samples were snap frozen in liquid nitrogen (for *in situ *TdT-mediated dUTP nick end labeling [TUNEL] and Western blot assays, and assessment of lung myeloperoxidase [MPO] activity) or embedded in paraffin (for histological examination); the remaining tissue was utilized for determination of lung wet/dry ratio (see below).

### Experimental protocol

After a 1 hour period of stabilization, cats were assigned to seven treatment groups (*n *= 5–6/group). The doses of the A_3_AR agonist IB-MECA [22,23,28,29] and antagonist [22,23,30,31] and their pretreatment times [22,23,31,32] were selected based on previous *in vivo *studies in cats, mice, rats, and rabbits.

#### Group i: nonischemic group

The LLL was perfused for 4 hours (no ischemia).

#### Group ii: ischemia/reperfusion group

Animals were subjected to ischemia and reperfusion of the LLL.

#### Groups iii, iv, and v: IB-MECA groups

The selective A_3_AR agonist IB-MECA, at doses of 50 μg/kg (group iii), 100 μg/kg (group iv), or 300 μg/kg (group v), was administered systemically as an intravenous bolus 1 hour after the beginning of the ischemic period.

#### Groups vi and vii: MRS3558 groups

The selective A3AR agonist MRS3558, at doses of 50 μg/kg (group vi) or 100 μg/kg (group vii), was administered systemically as an intravenous bolus 1 hours after the beginning of the ischemic period.

#### Group viii: MRS3558/MRS1191(A_3 _adenosine receptor agonist + antagonist) group

To ascertain whether MRS3558-induced modulation of lung injury, apoptosis, and MAPK activation is mediated by A_3_AR, in further studies the ability of an A_3_AR antagonist to block the effect of MRS3558 was evaluated. MRS3558 (100 μg/kg intravenously) was given systemically 1 hour after beginning of the ischemic period as in the other groups, with pretreatment 15 minutes earlier with the selective A_3_AR antagonist 3-ethyl-5-benzyl-2-methyl-4-phenylethynykyl-6-phenyl-1,4-(±)-dihydropyridine-3,5 dicarboxylate (MRS1191; 1 mg/kg intravenously).

### Assessment of injury and apoptosis

For light microscopy, samples of lung tissue were embedded in paraffin, cut into 4 μm slices, and stained with hematoxylin and eosin. The slides were coded and examined in a blinded manner by a single examiner. A total of 50 microscopic fields at ×40 magnification were examined in each section and the total number of alveoli in the 50 microscopic fields was scored. The severity of alveolar injury was assessed according to the percentage of injured alveoli, as defined previously [22,23,26,27]. Excised samples of lung tissue were also snap frozen in liquid nitrogen and stored at -70°C for determination of lung MPO [22,23]. The remainder of the left and right lower lobes was utilized for determination of lung wet/dry weights ratio, after sequential weights demonstrated maximal dehydration at 80°C.

Apoptosis was assessed using the TdT-mediated TUNEL assay, as described previously [22,23]. This was performed on formaldehyde-fixed lung sections using the Deadend Fluorometric TUNEL System (Promega, Madison, WI, USA), in accordance with the manufacturer's instructions. TUNEL-stained tissue sections were examined with a fluorescent confocal microscope. First, the propidium iodide (PI) staining (red) was examined through a 520 nm filter at a magnification of ×100. PI stained all nucleated cells in the same manner. The magnification was then increased to ×400 and a color photomicrograph was taken. The same area was then similarly examined for apoptotic staining (bright green), using a 590 nm filter at a magnification of ×400. Six randomly chosen fields were used for cell counts. PI-stained cells (representing the total number of cells: alive + necrotic + apoptotic cells) were counted first, followed by TUNEL-positive cells (representing the number of apoptotic cells). Only apoptotic cells that could clearly be identified as individual cells in the pulmonary parenchyma were counted. Alveolar macrophages, phagotized cells, or cells floating in the alveolar space were not included in the count.

Lungs were also tested after the reperfusion period for caspase 3 activity and expression, as outlined previously in detail [22,23]. Caspase is derived from a pro-enzyme at the onset of apoptosis and plays an important role in the final common pathway of programmed cell death. During IR injury, increased caspase 3 activity is indicative of increased programmed cell death signal. Briefly, to detect caspase 3 activity, 150 μg lysate from each lung was combined with fluorogenic caspase 3 substrate, diluted to 300 mg/l in caspase assay buffer (250 mmol/l PIPES, 50 mmol/l EDTA, 2.5% CHAPS, and 125 mmol/l DTT), and measured immediately on a fluorometer at an excitation wavelength of 400 nm and an emission wavelength of 505 nm. Measurements were repeated every 10 minutes for 1 hour, and the slope of fluorescent units per hour was calculated. Values were compared with known standards to determine enzymatic activity. To study apoptosis-related caspase expression levels by Western blotting, the homogenate was sonicated and centrifuged at 35,000 *g *for 15 minutes. A total of 100 μg protein was loaded onto a 10% SDS-PAGE for electrophoresis and then transferred onto a nitrocellulose membrane (Osmotics Inc., Westborough, MA, USA). The membrane was blocked with nonfat dry milk, and probed with the polyclonal antibody to active caspase 3 (1/1000; Santa Cruz Biochemical, Santa Cruz, CA, USA) for 8 hours at 40°C, and then incubated with a 1/1000 dilution of horseradish peroxidase-conjugated secondary antibody (Sigma). Hyperfilm ECL was exposed to blots treated with ECL solution, developed in a film processor, and scanned using a Molecular Dynamics 300A laser densitometer (Molecular Dynamics, Sunnyvale, CA, USA). Membranes were subsequently stripped (62.5 mmol/l Tris-HCl [pH 6.8], 20% SDS, 100 mmol/l β-mercaptoethanol) and n-probed for actin. To allow comparison between groups, data are expressed as percentage density of bands versus nonischemic (group i) lungs.

### Western blotting for JNK, p38, and ERK

Lung tissue samples were analyzed for levels of the activated, phosphorylated forms of JNK, p38, and ERK1/2 by Western blotting. Kits were purchased from Cell Signaling Technology (Beverly, MA, USA) and used in accordance with the manufacturer's protocol. Briefly, lungs specimens were snap frozen in liquid nitrogen. Protein (50 μg) from the 10,000 g supernatant of lung specimens was resolved on 12% SDS-PAGE and transferred to nitrocellulose membranes (Osmotics Inc.). The membrane was blocked with 5% nonfat dry milk and immunoblotted with specific antibodies for each of the phosphorylated forms of MAPK (1:200) or actin C-11 (Santa Cruz Biotechnology) for 8 hours at 40°C, and then incubated with a 1/1000 dilution of horseradish peroxidae-conjugated secondary antibody (Sigma) for 2 hours at room temperature. Quantitative analysis of the band density was performed using simultaneously blotted actin density to correct for protein content. Relative band intensities, expressed in arbitrary units of phospho-JNK, phospho-p38 and phospho-ERK to control (group i, no ischemia), were assessed by densitometry using a ChemiImager 4000 Imaging System (Alpha Innotech Corp., San Leandro, CA, USA).

### Statistical analysis

Data were analyzed using Student's *t* test when comparing means of two groups or with one-way analysis of variance with Bonferroni correction for multiple comparisons between groups. Differences were considered significant at *P *< 0.05. Results are expressed as mean ± standard error of the mean. Data were analyzed using SigmaStat (Jandel; San Rafael, CA, USA).

## Results

### Ischemia/reperfusion-induced lung injury and apoptosis

**Figure 1:**
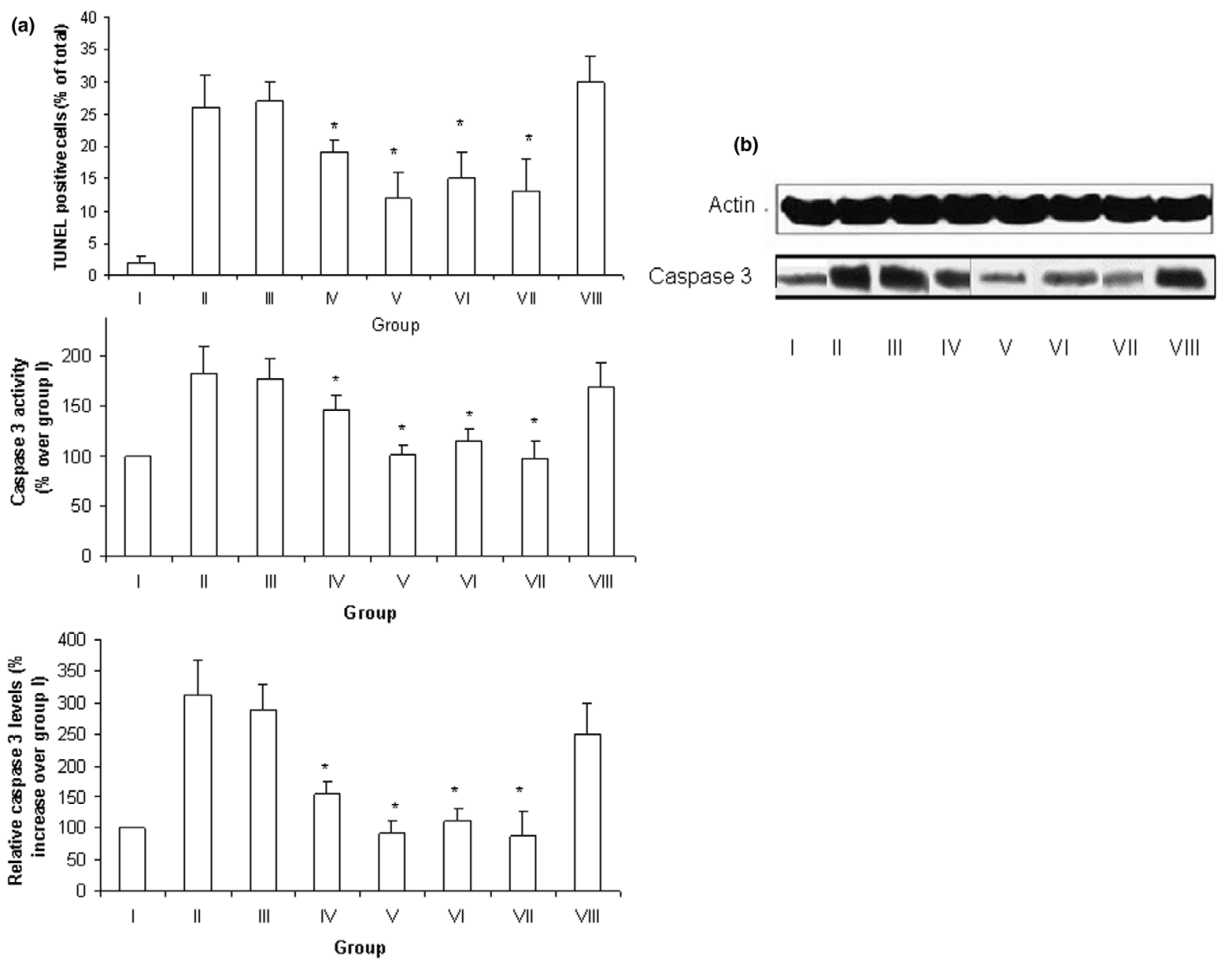
Parameters of apoptosis. **(a) **TdT-mediated dUTP nick end labeling (TUNEL)-positive cells (upper panel), changes in caspase 3 activity (middle panel), and changes in activated caspase 3 expression as analyzed by Western blotting (lower panel) in the left lower lobe of the different groups. Values are expressed as means ± standard error of the mean (*n *= 5–6 cats/group). **(b) **Representative Western blot analysis of caspase 3. The groups were as follows: i = nonischemic group; ii = ischemia/reperfusion; iii, iv and v = IB-MECA 50 μg/kg, 100 μg/kg and 300 μg/kg, respectively, administered 1 hour before reperfusion; vi and vii = MRS3558 50 μg/kg and 100 μg/kg, respectively, administered before reperfusion; and viii = MRS1191 pretreatment before MRS3558 (100 μg/kg) and beginning of reperfusion. *P < 0.05 versus the other groups (i, ii, iii, and viii).

**Figure 2:**
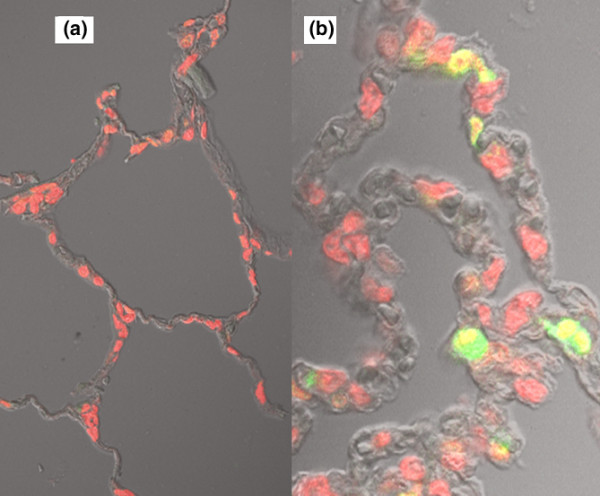
Representative results of TUNEL staining. Alveolar parenchyma from left lower lobe: **(a) **Nonischemic (group i) and **(b) **ischemia/reperfusion injury (group ii). Note that there are more bright green cells in panel b than in panel a, indicating more apoptosis. TUNEL, TdT-mediated dUTP nick end labeling.

Two hours of ischemia and 3 hours of reperfusion caused lung injury, manifested by a significant increase in the percentage of injured alveoli compared with the control group (no ischemia). Marked lung edema and inflammation, as assessed by wet/dry weight ratio of the LLL and by MPO activity, respectively, were also observed (Table 1). Examination of lung tissue revealed TUNEL-positive cells in the nonischemic lung as well as in lungs subjected to IR (Figures 1 and 2). Quantitative analysis revealed a significant increase in the number of TUNEL-positive cells in the IR lungs compared with nonischemic lungs (2 ± 1% and 26 ± 5%, respectively; *P *< 0.001). A change in expression of apoptosis-related caspase 3 protein during the course of IR was analyzed by Western blotting. Activated caspase 3 protein significantly increased after IR injury (312 ± 56% increase over group I [nonischemic]). Caspase 3 activity was also significantly increased in samples from the IR group (183 ± 27% increase over group i) compared with the nonischemic group.

### Comparative effects of the A_3 _adenosine receptor agonists on ischemia/reperfusion lung injury and apoptosis

The effects of the two selective A_3_AR agonists IB-MECA and MRS3558 on IR lung injury and apoptosis were evaluated and compared. Administration of both agonists before reperfusion attenuated injury (Table 1) and apoptosis (Figure 1). IB-MECA caused dose-related decreases in all parameters of injury. IB-MECA at 50 μg/kg had no significant effect on the mean percentage of injured alveoli, wet/dry weight ratio or MPO activity, but the higher doses (100 μg/kg and 300 μg/kg) significantly attenuated injury, with significant differences between the two doses. At the highest dose (300 μg/kg) the percentage of injured alveoli was reduced by 40%, and wet/dry weight ratio and MPO activity was nearly halved when compared with those in the IR group. With MRS3558 treatment no significant differences were found in the extent of attenuation of lung injury parameters between the two doses. Decreases in parameters of injury with MRS3558 50 μg/kg and 100 μg/kg were significantly greater than those observed with IB-MECA 50 μg/kg or 100 μg/kg. However, the two doses of MRS3558 attenuated injury to the same extent as did IB-MECA 300 μg/kg.

Activation of A_3_AR by IB-MECA and MRS3558 also caused significant attenuation in IR-induced apoptosis (Figure 1). Markers of apoptosis decreased by approximately 50% in the group receiving the higher dose of IB-MECA (300 μg/kg; group v) compared with the corresponding IR (group ii) values (group v versus group ii: percentage of TUNEL^+ ^cells 12 ± 4% versus 26 ± 5%; caspase 3 activity [versus that in group i] 101 ± 11% versus 183 ± 27%; expression levels of activated caspase 3 protein [versus that in group i] 91 ± 21% versus 312 ± 51%; *P *< 0.01). However, they did not achieve nonischemic values (group i). The magnitude of the effect of 300 μg/kg IB-MECA was similar to that observed with 50 μg/kg and 100 μg/kg MRS3558. The lower doses of IB-MECA had either reduced effect (100 μg/kg) or no effect at all (50 μg/kg) on indices of apoptosis. The lung protective effects of the A_3_AR agonists cannot be ascribed to the vehicle dimethyl sulfoxide (DMSO) because the same dose of DMSO had no effect on IR lung damage (data not shown).

To ascertain whether MRS3558 induced lung protection is mediated by A_3_AR, in further studies we evaluated the ability of an A_3_AR antagonist to block the effect of MRS3558 on IR lung injury. The highly selective A_3_AR antagonist MRS1191, given before MRS3558 (group viii), completely abolished the protection conferred by pretreatment with MRS3558; in this group lung injury parameters were indistinguishable from those measured in the IR group (group ii).

### Activation of mitogen-activated protein kinase after ischemia/reperfusion and following pretreatment with A_3 _adenosine receptor agonists

**Figure 3:**
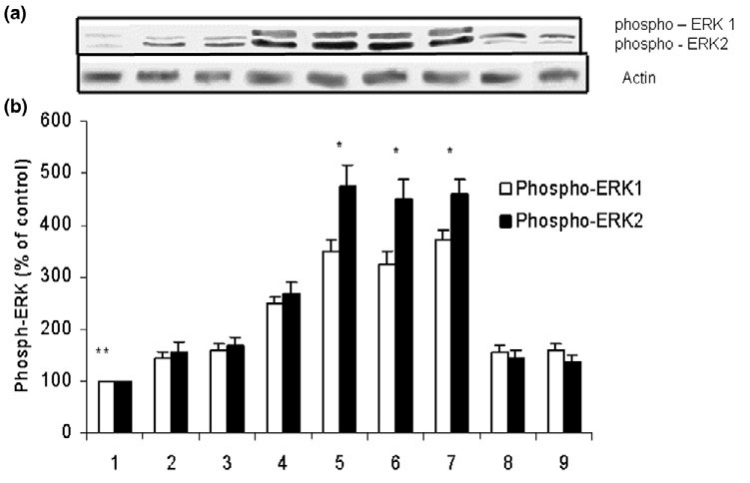
Western blot analyses of phospho-ERK1/2. **(a) **Representative Western blot and **(b) **densitometric analysis of five blots (mean ± standard error of the mean), expressed as a percentage of values for corresponding control-treated tissue samples (no ischemia or reperfusion). Groups: 1 = control, nonischemic group; 2 = ischemia/reperfusion; 3–5 = IB-MECA 50 μg/kg, 100 μg/kg, and 300 μg/kg, respectively, administered before reperfusion; 6 and 7 = MRS3558 50 μg/kg and 100 μg/kg, respectively, administered before reperfusion; 8 = MRS1191 pretreatment before MRS3558 (100 μg/kg) and beginning of reperfusion; and 9 = DMSO administration. Densitometry data presented is normalized to the intensity of actin bands. *P < 0.01 versus groups 1–4, 8, and 9. **P < 0.05 versus the other groups. DMSO, dimethyl sulfoxide; ERK, extracellular signal-regulated kinase.

**Figure 4:**
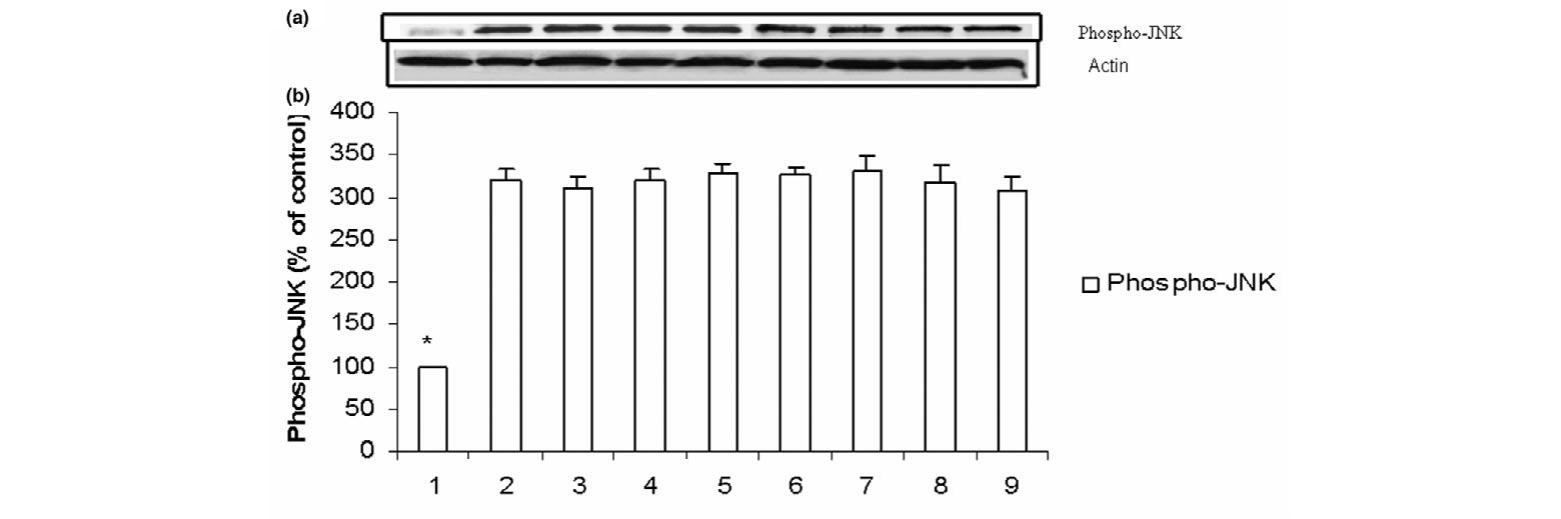
Western blot analyses of of phospho-JNK. **(a) **Representative Western blot and **(b) **densitometric analysis of five blots (mean ± standard error of the mean) expressed as a percentage of values for corresponding control-treated tissue samples (no ischemia or reperfusion). Groups: 1 = control, nonischemic group; 2 = ischemia/reperfusion; 3–5 = IB-MECA 50 μg/kg, 100 μg/kg, and 300 μg/kg, respectively, administered before reperfusion; 6 and 7 = MRS3558 50 μg/kg and 100 μg/kg, respectively, administered before reperfusion; 8 = MRS1191 pretreatment before MRS3558 (100 μg/kg) and beginning of reperfusion; and 9 = DMSO administration. Densitometry data presented is normalized to the intensity of actin bands. *P < 0.001 versus the other groups. DMSO, dimethyl sulfoxide; JNK, c-Jun amino-terminal kinase.

**Figure 5:**
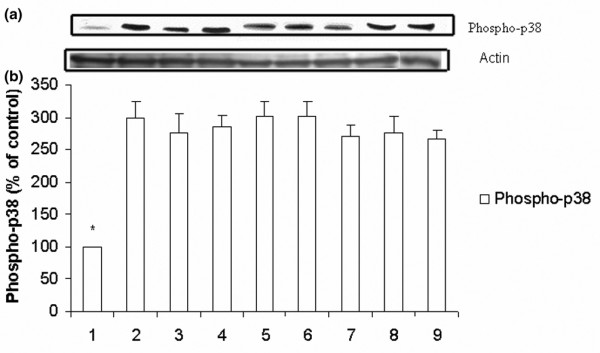
Western blot analyses of phospho-p38. **(a) **Representative Western blot and **(b) **densitometric analysis of five blots (mean ± standard error of the mean) expressed as a percentage of values for corresponding control-treated tissue samples (no ischemia or reperfusion). Groups: 1 = control, nonischemic group; 2 = ischemia/reperfusion; 3–5 = IB-MECA 50 μg/kg, 100 μg/kg, and 300 μg/kg, respectively, administered before reperfusion; 6 and 7 = MRS3558 50 μg/kg and 100 μg/kg, respectively, administered before reperfusion; 8 = MRS1191 pretreatment before MRS3558 (100 μg/kg) and beginning of reperfusion; and 9 = DMSO administration. Densitometry data presented is normalized to the intensity of actin bands. * *P *< 0.001 versus the other groups. DMSO, dimethyl sulfoxide.

To determine the role, if any, of MAPK in lungs after IR, we performed Western blot studies with specific phospho-antibodies for ERK1/2, JNK, and p38 MAPK. As seen in Figures 3, 4, 5, phosphorylated ERK1/2, JNK, and p38 were all increased by the end of reperfusion compared with controls, although the increase in the latter two MAPK members was significantly greater than that for ERK1/2.

Pretreatment with the A_3_AR agonists, at all doses, did not cause any change in levels of phosphorylated JNK and p38. However, significant upregulation in the level of phosphorylated ERK1/2 level was found at the end of reperfusion with the higher doses of IB-MECA (100 μg/kg and 300 μg/kg) and the two doses of MRS3558 (50 μg/kg and 100 μg/kg) compared with IR and control, and acute administration of DMSO (group ix; Figures 3, 4, 5). No significant differences in activated ERK1 and ERK2 levels were observed between the two doses of MRS3558. Administration of the selective A_3_AR antagonist before the A_3_AR agonist (MRS3558) completely abolished the upregulation of phosphorylated ERK1/2 observed with MRS3558, and had no effect on the upregulation of phospho-JNK and phospho-p38 that was observed with reperfusion.

In none of the groups was acidosis, significant increase in partial carbon dioxide tension, or decrease in partial oxygen tension observed during ischemia or reperfusion (data not shown).

## Discussion

We previously showed that administration of IB-MECA, an A_3_AR agonist, during lung reperfusion attenuates injury and apoptosis [22,23]. Given the important role played by MAPK in reperfusion injury [3,4,7,33], the aim of the present study was to determine whether A_3_AR-induced lung protection is mediated by modulation of MAPK members. Using an *in vivo *model of IR lung injury, our study shows for the first time that stimulation of A_3_AR activates ERK1/2 during reperfusion, suggesting the involvement of ERK1/2 in A_3_AR-mediated lung protection. The present study also demonstrates that levels of phosphorylated JNK, p38, and ERK1/2 are increased following reperfusion compared with nonischemic lungs (controls). The increases in expression of phosphorylated JNK and p38 after 3 hours of reperfusion were significantly greater than that in phosphorylated ERK1/2. In addition, we found the newly synthesized (*N*)-methanocarba A_3_AR analog MRS3558 to be more potent than IB-MECA in attenuating reperfusion lung injury and apoptosis.

Previous studies reported a role for MAPK in mediating the pathologic sequelae of cardiac, hepatic, and renal reperfusion injury [7]. ERK, JNK, and p38 kinases were shown to be activated in lung reperfusion injury, but studies in different animal models reported conflicting results. Sakiyama and coworkers [34] found a lack of p38 activation with reperfusion of rat lung grafts. Using type II rat pneumocytes, 2 hours of hypoxia followed by 4 hours of reoxygenation failed to activate p38 or JNK, whereas activation of ERK1/2 was observed [35]. In isolated perfused rat lungs, levels of expression of phosphorylated JNK, p38, and ERK1/2 were enhanced during reperfusion [10]. Similarly, in pig lung following cardiopulmonary bypass, expression of p38 and ERK1/2 has been demonstrated [33]. Our results are in agreement with the latter studies [10,33]; activation of the three MAPKs was identified after 3 hours of reperfusion, although to different extents. Because we measured phosphorylated ERK only at the end of reperfusion, it could also be – as suggested in previous studies [36] – that there was an early activation of ERK during ischemia and/or reperfusion, with a subsequent decline. Other possible causes of the observed conflicting results between studies include species differences and use of different experimental models.

In the current medical literature on management of IR syndromes, MAPKs are emerging as important targets for study. It has been noted that whereas ERK1/2 exerts a cytoprotective effect and is involved in cell proliferation, transformation, and differentiation, p38 and JNK promote cell injury and apoptosis [4,7,11,37,38]. Previous studies showed that appropriate inhibition of MAPK could control and ameliorate the reperfusion response. For example, suppression of p38 activation has been reported to attenuate injury in a warm IR rat model [8] and in canine lung transplantation-related IR [9]. Similarly, inhibiting JNK activity during periods of both ischemia and reperfusion attenuated lung injury and apoptosis caused by IR in isolated perfused rat lungs [10]. In contrast, pharmacologic inhibition of ERK enhanced IR-induced apoptosis in cultured cardiac myocytes and exaggerated reperfusion injury in isolated perfused heart [11]. In reperfused lung, the present study shows that A_3_AR activation upregulates phosphorylated ERK but not phosphorylated p38 and JNK, resulting in attenuation of apoptosis and injury. Because levels of the MAPK members were only measured after 3 hours of reperfusion, we cannot rule out the possibility that there is only a shift in the timing of activation of ERK1/2 compared with JNK and p38 kinase, and not an absolute difference in the level of their activation in response to A_3_AR activation.

Adenosine has been found to activate ERK1/2 in perfused rat heart [39,40] and to exert delayed preconditioning in a rat IR heart model by MAPK-dependent mechanisms [14,15]. In the present study MAPKs were activated during reperfusion (although for ERK this was to a significantly lesser extent), suggesting that in the reperfused lung both aggravating signals (related to either JNK or p38) and protective signals (related to ERK) interact in a complicated manner. Although the combined activities of these MAPKs resulted in injury and apoptosis, we observed improvement in injury and attenuation of apoptosis with A_3_AR-induced ERK activation (Table 1 and Figure 1). Whether ERK1/2 activation is essential for the observed A_3_AR-induced lung protection cannot be determined from the present study because experiments with a ERK1/2 blocker were not conducted. Preliminary data from our laboratory (data not shown) with the specific inhibitor of MAPK/ERK1/2 (MEK1/2) suggest that there was enhancement in lung injury following reperfusion in animals not treated with the A_3_AR agonists, emphasizing the critical role played by ERK1/2 in tissue protection. These results are in agreement with those from an earlier study [11], which found that ERK activation prevented the cardiomyocytes from apoptosis during reoxygenation when the JNK and p38 were activated. As in the present study, ERK activation could not totally prevent reoxygenation-induced myocyte apoptosis [11]. Although not investigated in the present study, in cardiomyocytes the signaling mechanisms implicated in ERK1/2 activation by A_3_AR involve adenylyl cyclase activation via phospholipase C and protein kinase C stimulation [39]. Also, activation of ERK subsequent to stimulation of P2Y6 nucleotide receptors by UDP was found to attenuate tumor necrosis factor-α-induced apoptosis [41].

**Figure 6:**
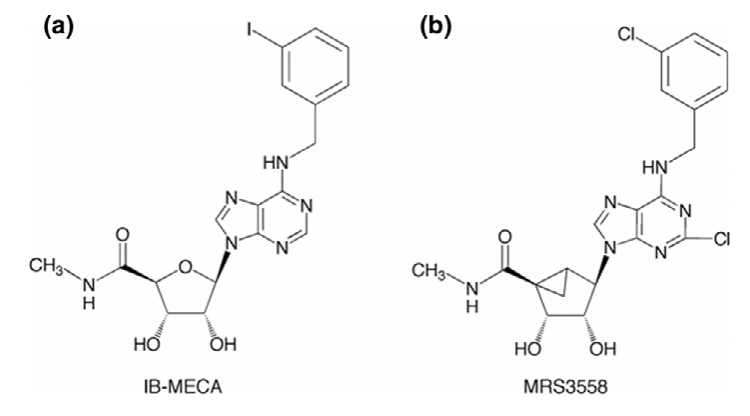
Structures of the two A_3_AR-selective agonists used in the study. **(a) **IB-MECA is a 9-riboside derivative, a structural feature shared with native adenosine. **(b) **The more potent and selective agonist MRS3558 contains a modified methanocarba ring system in place of ribose. The methanocarba substitution consists of fused cyclopentane and cyclopropane rings, which serve to constrain this moiety in a conformation that is preferred in the A_3_AR binding site. Both analogs share the N6-benzyl-type substitution, which is particularly suited for high affinity at the A_3_AR in various species. The methylamide moiety attached at the 4'-position serves to increase A_3_AR affinity and to maintain full efficacy in A_3_AR activation. The 2-chloro substitution of MRS3558 enhances A_3_AR affinity and selectivity. AR, adenosine receptor.

IB-MECA is a moderately A_3_AR-selective nucleoside that is a full agonist [42] (Figure 6). Previous reports have indicated limited pharmacologic selectivity of IB-MECA [43,44] and suggest that there is a need for development of more selective A_3_AR agonists. The principal element of the recently designed, highly potent and selective A_3_AR agonists is a modified ribose moiety. The analogs contain the (*N*)-methanocarba ring system, which is a rigid ribose substitute that lacks the ether oxygen. This ring system maintains the 2'-exo-(*N*) ring-twist conformation of the ribose-like ring (pseudosugar moiety), which has been demonstrated to be favored in A_3_AR binding (more so than at other AR subtypes). These agonists also contain a flexible 5'-uronamide group, which is necessary for full activation of the A_3_AR.

In the present study we evaluated MRS3558 [25], which belongs to the (*N*)-methanocarba series, and found that this agent is more potent than IB-MECA in attenuating IR lung injury and apoptosis. In previous studies, the highly selective A_3_AR antagonist MRS1191 abolished the lung protection provided by IB-MECA [22,23]. Similarly, MRS1191 inhibited MRS3558 lung protective effects, confirming that it acts through A_3_AR. The enhanced potency of MRS3558 in cat was in agreement with the relative binding affinity measured at the human A_3_AR [25]. This is the first application of this novel agonist *in vivo*, and we have demonstrated its efficacy in activating the A_3_AR. Further experiments will be required to determine the pharmacokinetic characteristics of MRS3558, but it may prove to be more stable *in vivo *than riboside-based agonists because of the presence of the methanocarba moiety. Based on calculated logP values and its having a lower molecular weight and fewer H-bond acceptors [45], MRS3558 would appear to be more 'drug-like' than IB-MECA. These log *P *values are 2.02 for MRS3558 and 0.48 for IB-MECA, and the molecular weights are 463 and 510, respectively. The determination of the precise *in vivo *AR selectivity of MRS3558 is beyond the scope of the present study; however, at the doses administered, it did not cause a reduction in heart rate or peripheral blood pressure, indicating that the state of activation of A_1_AR and A_2A_AR were unaffected. This degree of selectivity is consistent with reported binding affinities at human ARs.

Experiments conducted in the present study were performed in an *in vivo *model in which the LLL of the lung was isolated and subjected to 2 hours of ischemia and 3 hours of reperfusion. However, the isolated lobe was perfused with nonpulsatile flow, and therefore these experiments may not fully represent the *in vivo *pulmonary milieu. Previous studies have documented lower pulmonary vascular resistance in lung preparations perfused by pulsatile versus steady flow [46-50], possibly mediated by several different mechanisms including endothelial-dependent nitric oxide release, passive recruitment of capillaries, and active vasodilatation [51-53]. Moreover, pulsatile flow has been shown to affect neutrophil sequestration in the lung, although both increases and decreases in pulmonary sequestration were reported with the use of pulsatile flow compared with nonpulsatile flow [50,54].

## Conclusion

Using an *in vivo *model of IR lung injury, we found that levels of phosphorylated JNK, p38, and ERK1/2 are increased following reperfusion. The increased expression of phosphorylated JNK and p38 after 3 hours of reperfusion was significantly greater than that with phosphorylated ERK1/2. A_3_AR activation significantly upregulated ERK1/2 expression, leading to marked improvement in lung injury and attenuation of apoptosis after reperfusion. The newly synthesized (*N*)-methanocarba A_3_AR analog MRS3558 was more potent than IB-MECA in attenuating lung injury and apoptosis. Our findings suggest not only that enhancement of the ERK pathway may shift the balance between cell death and survival toward cell survival, but also that use of the A_3_AR agonists is potentially an effective therapeutic strategy against IR-induced lung injury. Further investigation is necessary to determine the precise signaling mechanisms implicated in ERK1/2 activation by A_3_AR.

## Key messages

• 	Levels of phosphorylated JNK, p38, and ERK1/2 levels are increased following 2 hours of ischemia and 3 hours of reperfusion.

• 	The increase in the expression of phosphorylated JNK and p38 after 3 hours of reperfusion is significantly greater than that of phosphorylated ERK1/2.

• 	Administration of A_3_AR agonists before reperfusion attenuated lung injury and apoptosis.

• 	Activation of A_3_AR activates ERK1/2 during reperfusion, suggesting the involvement of ERK1/2 in A_3_AR-mediated lung protection.

• 	The newly synthesized (*N*)-methanocarba A_3_AR analog MRS3558 is more potent than IB-MECA in attenuating reperfusion lung injury and apoptosis.

## Abbreviations

AR = adenosine receptor; DMSO = dimethyl sulfoxide; ERK = extracellular signal-regulated kinase; IR = ischemia/reperfusion; JNK = c-Jun amino-terminal protein kinases; LLL = left lower lobe; MAPK = mitogen activated protein kinases; MPO = myeloperoxidase; PI = propidium iodide; TUNEL = *in situ *TdT-mediated dUTP nick end labelling.

## Competing interests

The authors declare that they have no competing interests.

## Authors' contributions

IM and KAJ conducted the experiments and participated in the design of the study. IM also analyzed the data and drafted the manuscript, while KAJ suggested improvements to the manuscript. EZ performed all assays of lung injury and apoptosis under the supervision of EG. CW performed surgical procedures under the supervision of IM. BVJ and KAJ synthesized the A_3_AR agonist. All authors read and approved the final manuscript.

## Figures and Tables

**Table 1:** Lung injury following ischemia and reperfusion

Group	% injured alveoli	MPO activity^a^	Wet/dry weight ratio
i	2.8 ± 1.3*	1.4 ± 0.3*	4.8 ± 0.4
ii	35.0 ± 2.2	4.7 ± 0.3	8.6 ± 0.3
IB-MECA groups			
iii (50 μg/kg)	32.5 ± 2.3**	4.4 ± 0.3**	8.0 ± 0.3**
iv (100 μg/kg)	27.7 ± 1.4^# ^**	3.6 ± 0.2^# ^**	6.4 ± 0.3^# ^**
v (300 μg/kg)	21.0 ± 1.5^#^	2.3 ± 0.2^#^	5.2 ± 0.2^#^
MRS3558 groups			
vi (50 μg/kg)	22.2 ± 1.5^#^	2.4 ± 0.3^#^	5.3 ± 0.4^#^
vii (100 μg/kg)	20.1 ± 2.2^#^	2.0 ± 0.2^#^	4.9 ± 0.3^#^
viii (MRS3558-MRS1191 group)	37.7 ± 1.5	4.8 ± 0.3	8.5 ± 0.3

Values are expressed as means ± standard error of the mean (*n *= 5–6 cats/group). IB-MECA and MRS3558 are A_3 _adenosine receptor (AR) agonists, and MRS1191 is an A_3_AR antagonist. The groups were as follows: i = nonischemic group; ii = ischemia/reperfusion; iii, iv and v = IB-MECA 50 μg/kg, 100 μg/kg and 300 μg/kg, respectively, administered 1 hour before reperfusion; vi and vii = MRS3558 50 μg/kg and 100 μg/kg, respectively, administered before reperfusion; and viii = MRS1191 pretreatment before MRS3558 (100 μg/kg) and beginning of reperfusion. ^a^Tissue myeloperoxidase (MPO) activity is expressed in units of MPO/g of lung weight, each of which was defined as the activity degrading 1 μmol of peroxide per minute at 25°C. *P < 0.01 versus the other groups; **P < 0.05 versus MRS3558 groups and IB-MECA 300 μg/kg group; ^#^*P *< 0.05 versus IR and MRS3558-MRS1191 groups (see text for further comparisons).
